# Association between physical activity and inflammatory bowel disease: a prospective cohort study

**DOI:** 10.3389/fpubh.2026.1819729

**Published:** 2026-05-14

**Authors:** Yitian Wei, Binbin He, Ge Zhang, Haitao Chen, Tuojian Li

**Affiliations:** 1 School of Physical Education, Shandong University, Jinan, China; 2 School of Public Health, Sun Yat-sen University, Shenzhen, China

**Keywords:** Crohn's disease, exercise intensity, inflammatory bowel disease, physical activity, UK Biobank, ulcerative colitis

## Abstract

**Objective:**

Evidence regarding the association between physical activity (PA) and the long-term incidence of inflammatory bowel disease (IBD) remains limited and inconsistent. This study aimed to evaluate the longitudinal relationship between varying intensities of PA and the risk of developing IBD, specifically Crohn's disease (CD) and ulcerative colitis (UC).

**Methods:**

This prospective cohort study analyzed data from 293,578 participants enrolled in the UK Biobank. Self-reported PA was categorized by intensity into light, moderate, and vigorous levels. Incident IBD cases were identified through linked hospital admission and death registry records. The association between PA levels and IBD incidence was assessed using multivariable Cox proportional hazards regression models. Furthermore, restricted cubic spline curves were utilized to examine potential non-linear associations, and cumulative event curves were generated to visualize disease progression over time.

**Results:**

During the follow-up period, higher levels of total PA were significantly associated with a reduced risk of CD, suggesting a protective effect of consistent physical exertion. While the relationship between PA and UC was insignificant in the general cohort, a significant reduction in UC risk was observed among individuals whose activity levels exceeded standard health recommendations. Notably, the data revealed a complex intensity-dependent relationship: whereas moderate-to-vigorous activity generally trended toward benefit, excessive light PA was paradoxically associated with an increased risk of UC within specific subpopulations.

**Conclusion:**

In this large-scale prospective study, PA was inversely associated with the risk of CD. However, the association between PA and UC appears more nuanced, with the protective benefits potentially contingent upon higher activity thresholds and specific intensity profiles. These findings suggest that tailored exercise recommendations may be necessary for IBD prevention strategies.

## 1 Introduction

Inflammatory bowel disease (IBD) encompasses a group of chronic, idiopathic gastrointestinal disorders characterized by persistent inflammation of the digestive tract, with ulcerative colitis (UC) and Crohn's disease (CD) representing the two primary clinical phenotypes. While both conditions share common inflammatory pathways, they are distinguished by their anatomical distribution and histological depth. CD is characterized by discontinuous and transmural (full-thickness) inflammation that can affect any segment of the gastrointestinal tract, from the mouth to the anus. In contrast, UC is typically restricted to the colon and rectum, characterized by continuous inflammation predominantly localized to the mucosa and submucosa (1). The global incidence of IBD is rising at an accelerating rate, particularly within industrialized nations. The disease most frequently manifests in young adulthood, often resulting in a lifelong clinical course marked by unpredictable, recurrent exacerbations. This chronicity exerts a profound impact on patients' quality of life, manifesting in significant physical morbidity and emotional distress. Current epidemiological data indicate that the global incidence of CD has reached 23 per 100,000 person-years, while the incidence of UC is 57 per 100,000 (2). Given this escalating prevalence and the substantial burden on healthcare systems, IBD has emerged as a critical global health priority necessitating expanded investigative efforts into modifiable risk factors (2).

Physical activity (PA) is defined as any bodily movement produced by skeletal muscles that results in energy expenditure. It is a cornerstone of metabolic and cardiovascular health, widely recognized for its role in the primary prevention and management of chronic diseases, the enhancement of cognitive function, and the promotion of psychological wellbeing (3). Conversely, physical inactivity is a well-established risk factor for a spectrum of non-communicable diseases and all-cause mortality. In the context of IBD, exercise—a subcategory of PA that is planned, structured, and repetitive—has garnered significant interest due to its potent systemic anti-inflammatory effects. This exercise-induced anti-inflammation is largely mediated through the release of myokines, such as interleukin-6, from contracting skeletal muscles, which subsequently stimulates an increase in anti-inflammatory cytokines such as interleukin-10. As IBD patients increasingly pursue holistic management strategies to complement conventional pharmacotherapy and surgical interventions, PA has emerged as a promising adjunctive therapy. Preliminary evidence suggests that regular PA may not only reduce the severity of symptoms but also improve bone mineral density and psychological resilience in this patient population (4). Despite these potential benefits, the precise dose-response relationship between PA intensity and the primary prevention of CD and UC remains insufficiently characterized.

Despite the theoretical benefits of exercise, empirical evidence regarding how the intensity, duration, and type of PA influence IBD risk and morbidity remains fragmented. Existing literature is frequently constrained by modest sample sizes or narrow demographic cohorts, often limiting the generalizability of the findings. For instance, a large-scale Danish cohort study reported no significant association between PA intensity or duration and IBD risk specifically among older adults (5). In contrast, cross-sectional data from Australia utilizing objective accelerometer measurements demonstrated that patients with CD were significantly less active over a seven-day period compared to healthy controls, though this study was limited by a small sample size (*n* = 79). Evidence regarding disease-specific outcomes also remains inconsistent. An analysis of two large prospective cohorts of American women found that while PA was inversely associated with the risk of CD, no such protective effect was observed for UC (6). This discrepancy is mirrored in secondary research; a meta-analysis encompassing two cohort and five case-control studies identified a significant inverse relationship between PA and CD risk, yet found no correlative benefit for UC. Recent findings by Zhao et al. (7) further corroborate this divergence, reinforcing the role of PA as a protective factor specifically for CD. These disparate findings underscore a critical need for high-powered, longitudinal research. Specifically, there is a requirement to elucidate the dose-response relationship between different PA intensities—light, moderate, and vigorous—and the distinct clinical trajectories of CD and UC.

Recent investigations have increasingly elucidated the therapeutic potential of physical exercise in the management of IBD. Beyond its systemic benefits, regular exercise has been shown to directly attenuate IBD symptomology and systemic inflammation, thereby significantly improving patient-reported quality of life (8). Furthermore, a comprehensive review by Engels et al. (9) reinforces the clinical consensus that exercise is both safe and efficacious for IBD patients. Their findings highlight the dual role of exercise in stabilizing disease activity and mitigating the psychological comorbidities—such as anxiety and depression—often associated with chronic gastrointestinal disorders. However, while the safety profile of exercise is well-established, the underlying biological mechanisms and the precise “prescription” (including the optimal intensity and volume of exercise) remain inadequately defined. Consequently, there is a clear imperative for large-scale, prospective data to delineate how specific activity thresholds influence disease risk. The present study seeks to address this gap by leveraging the UK Biobank cohort to investigate the association between various PA intensities and the long-term incidence of CD and UC.

To address the current gaps in epidemiological evidence, this study utilized a large-scale prospective design involving 294,288 participants from the UK Biobank. By using this extensive longitudinal dataset, we aimed to rigorously analyze the PA patterns and intensities—stratified into light, moderate, and vigorous levels—and determine their subsequent impact on the incidence of IBD.

The primary objective was to evaluate the association between PA and IBD risk, with a specific focus on identifying how distinct activity intensities differentially influence the development of CD and UC. Furthermore, this research sought to establish evidence-based recommendations for IBD prevention, providing clinical insights into how specific PA patterns may be utilized as a modifiable strategy to reduce disease burden.

## 2 Methods

### 2.1 Study participants

The UK Biobank is a large biomedical cohort study database established by the United Kingdom government. Since 2006, in-depth genetic and phenotypic data have been collected from approximately 500,000 individuals across the United Kingdom, aged 40–69 years at recruitment. For all participants in our study, data analyzed included the first available physical examination (“baseline”). Prospective analyses were conducted on participants from baseline to their last examination, provided they had at least two assessments and met one of the following criteria: (i) no IBD at baseline, and (ii) available data on PA and other covariates. Follow-up for participants began at their first UK Biobank visit and continued until the occurrence of one of the following: a diagnosis of CD or UC, death, immigration, or September 30, 2022, whichever came first. To ensure an accurate analysis of the relationship between PA and IBD, we excluded individuals with missing crucial data (such as information on PA, smoking, alcohol consumption, or inconsistent gender) from the original cohort of 502,368 individuals with questionnaire measurements. Additionally, we excluded data from 1,261 CD participants to 2,284 UC participants who already had IBD at baseline. After these exclusions, a total of 293,578 participants remained in this study.

### 2.2 IBD assessment

IBD in the UK Biobank was identified through hospital admission and death records. The date and reason for admission were determined by linking records with Hospital Episode Statistics (England and Wales) and Scottish Morbidity Records (Scotland). Data on the date and cause of death were obtained from the Death Register system of the National Health Service Information Center (England and Wales) and the National Health Service Central Register (Scotland). For more information, please visit http://www.ukbiobank.ac.uk/enable-your-research/. In this study, IBD was defined using ICD-10 codes (K50, including all subcodes, and K51, including all subcodes). Due to variations in the frequency and intervals of follow-up visits among participants, the end point for patients with IBD was defined as the time point at which IBD was first diagnosed during the follow-up period. Based on the hospital patient data from the UK Biobank, this study conducted a review of participants, examining their IBD status up to the last follow-up date (October 31, 2022). The date and year of the initial diagnosis were based on the first registration of the diagnosis in the UK Biobank system.

### 2.3 PA assessment

Data on PA were collected through self-reports in a questionnaire survey to validate the International Physical Activity Questionnaire (IPAQ). The questionnaire assessed participants' engagement in various physical activities, including leisure-time activities (e.g., climbing stairs, recreational activities, and walking), moderate-intensity activities, and vigorous-intensity activities. It also measured the frequency (number of days per week) and duration (minutes per day) of participants' involvement in these activities, as well as their total Metabolic Equivalent of Task (MET)-minutes of PA. One MET is defined as 1 kcal/kg/hour (1 large calorie consumed per kilogram of body weight per hour), which is approximately equivalent to the resting metabolic rate.

The PA-related data used in the UK Biobank includes a category that derives MET scores based on IPAQ guidelines. According to the IPAQ guidelines for data processing and analysis, three levels of MET values are defined: walking (light intensity) = 3.3 METs, moderate intensity = 4.0 METs, and vigorous intensity = 8.0 METs. Therefore, the MET-minutes data for participants in the MET scores category were calculated as follows:

- Walking MET-minutes/week = 3.3 ^*^ walking minutes ^*^ walking days.
- Moderate MET-minutes/week = 4.0 ^*^ moderate-intensity activity minutes ^*^ moderate days.
- Vigorous MET-minutes/week = 8.0 ^*^ vigorous-intensity activity minutes ^*^ vigorous-intensity days.
- Total PA MET-minutes/week = sum of Walking, Moderate, and Vigorous MET-minutes/week scores.

The World Health Organization's PA guidelines recommend that adults engage in at least 150 min of light-intensity PA and 150 min of moderate-intensity activity, or at least 75 min of vigorous-intensity aerobic activity per week. For additional health benefits, it is recommended to engage in more than 300 min of light and moderate PA or more than 150 min of vigorous-intensity PA per week. Furthermore, moderate-intensity and vigorous-intensity activities should be appropriately balanced. Based on the MET scores calculated according to the IPAQ guidelines, adults should aim to achieve 495 MET-minutes of light PA and 600 MET-minutes of moderate and vigorous PA per week. To gain additional health benefits, adults should aim for 990 MET-minutes of light PA and 1,200 MET-minutes of moderate and vigorous PA per week. The time distinctions for PA considered in this study are based on these standards.

### 2.4 Covariates

Based on known and generally accepted risk and preventive factors for IBD, the covariates considered in this study included age (<45, 45–60, and >60 years), gender, smoking status (never, past, or current), alcohol consumption status (never, former, or current), overall health (excellent, good, fair, or poor), and body mass index (BMI, calculated as weight in kilograms divided by height squared in meters, following the World Health Organization (WHO) criteria, and categorized as normal, overweight, or obese). These covariates were measured at the time of data collection.

### 2.5 Statistical analysis

The time calculation for the research participants was defined as the period from the date of returning the baseline questionnaire to the diagnosis of UC or CD, or to the date of the last returned questionnaire. The Cox proportional hazards model was used to explore the hazard ratios (HRs) between the risk of IBD events and PA, along with the corresponding 95% confidence intervals (CIs) and *p*-values. Both univariate and multivariate Cox proportional hazards regression analyses were conducted to identify the characteristics associated with the prognosis of the participants.

The cumulative incidence curve of events was used to assess the cumulative risk of different intensities of PA on the incidence of IBD events. The cumulative incidence curve, which changes over time, provides a more intuitive representation. In this case, the outcome of the curve represents the absence of any IBD events. A total of 1,826 participants with IBD were included in the analysis. The intensity and duration of the physical activities of these participants were further classified into the following categories: (i) not meeting the WHO-recommended PA standards, (ii) engaging only in light/moderate/high-intensity PAs, (iii) engaging only in light and moderate activities, (iv) engaging only in light and high-intensity activities, (v) engaging only in moderate and high-intensity activities, and (vi) a combination of all three intensities. The restricted cubic spline curve (RCS, truncated at the 5th, 25th, 50th, and 75th percentiles) was used to analyze the nonlinear relationship between different intensities of PA and the incidence of IBD. To obtain more reliable results, subgroup analyses were performed to examine whether the association differed by gender (male or female) or smoking status (never smokers and current/former smokers). To assess the robustness of the study, a sensitivity analysis was conducted. Initially, the model was adjusted multiple times by adding or removing covariates, resulting in two models: (i) excluding individuals with excessively high or low BMI (BMI <18.5 or BMI > 30) to account for the impact of abnormal body weight on the prevalence of IBD and PA, and (ii) limiting the analysis to IBD events that occurred at least 3 years after baseline, to minimize the possibility of reverse causality and reduce bias.

All analyses were conducted using R version 4.4.2 (R Core Team, Vienna, Austria). A *p*-value of less than 0.05 was considered statistically significant for all tests.

## 3 Results

After excluding participants who had IBD at baseline (*n* = 3,545) and those with missing information on all covariates, a total of 293,578 individuals were included in the analysis. At baseline, the mean (SD) age of the participants was 55.70 (8.07) years. Among the participants, 150,483 were female (51.2%) and 143,095 were male (48.8%). A total of 1,707 participants were diagnosed with IBD, including 597 with CD and 1,220 with UC. Additionally, 110 patients had both CD and UC simultaneously.

### 3.1 Associations of PA and other factors with IBD incidence

According to Tables 1, 2, significant differences were observed in the risk factors for CD and UC, as well as in the protective effect of PA. For CD, smoking and poor health status were the primary risk factors, with the risk significantly increased in current smokers (HR = 1.52, *p* < 0.001). Although vigorous PA showed a protective effect on CD, the effect was relatively limited, with only a slight protective benefit observed when the recommended PA duration set by the WHO was achieved (HR = 0.77, *p* = 0.028). Furthermore, engaging in more total PA per week appeared to have a positive effect in reducing the risk of CD (HR = 0.78, *p* = 0.017), though this effect was not significant after adjustment.

**Table 1:** Cox regression analysis of PA and incidence of CD.

Variables	Univariate analysis		Multivariate analysis	
	HR (95% CI)	*p*	HR (95% CI)	*p*
Age (years)				
<45	Reference		Reference	
45–65	0.77 (0.61–0.97)	0.024	0.75 (0.59–0.94)	0.014
>60	0.94 (0.75–1.2)	0.639	0.9 (0.7–1.15)	0.392
Sex				
0	Reference			
1	1.08 (0.92–1.26)	0.363		
Smoking status				
Never	Reference		Reference	
Former	1.38 (1.16–1.64)	<0.001	1.3 (1.09–1.56)	0.004
Current	1.86 (1.46–2.36)	<0.001	1.52 (1.19–1.95)	<0.001
Alcohol use status				
Never	Reference			
Former	1.30 (0.76–2.20)	0.339		
Current	0.73 (0.49–1.09)	0.123		
Overall health				
Excellent	Reference		Reference	
Good	1.91 (1.44–2.54)	<0.001	1.84 (1.38–2.46)	<0.001
General	3.40 (2.52–4.60)	<0.001	3.09 (2.25–4.25)	<0.001
Poor	5.66 (3.87–8.27)	<0.001	5.14 (3.42–7.72)	<0.001
BMI	1.03 (1.01–1.04)	0.001	1.00 (0.98–1.02)	0.982
Weekly light PA				
None or less than RT	Reference			
RT	1.04 (0.85–1.27)	0.702		
More than RT	0.98 (0.81–1.18)	0.815		
Weekly moderate PA				
None or less than RT	Reference			
RT	0.95 (0.77–1.17)	0.639		
More than RT	0.92 (0.75–1.13)	0.446		
Weekly vigorous PA				
None or less than RT	Reference		Reference	
RT	0.77 (0.61–0.97)	0.028	0.97 (0.73–1.29)	0.844
More than RT	0.84 (0.67–1.05)	0.123	1.09 (0.82–1.44)	0.546
Total weekly PA				
None or less than RT	Reference		Reference	
RT	0.91 (0.71–1.16)	0.435	1.02 (0.79–1.32)	0.855
More than RT	0.78 (0.64–0.96)	0.017	0.98 (0.74–1.29)	0.873
Above moderate/vigorous RT				
No	Reference		Reference	
Yes	0.83 (0.70–0.97)	0.019	0.93 (0.71–1.22)	0.5934

CI, confidence interval; HR, hazard ratio; RT, PA duration recommended by the WHO.

**Table 2:** Cox regression analysis of PA and incidence of UC.

Variables	Univariate analysis		Multivariate analysis	
	HR (95% CI)	*p*	HR (95% CI)	*p*
Age (years)				
<45	Reference		Reference	
45–65	1.00 (0.83–1.19)	0.966	0.97 (0.81–1.16)	0.733
>60	1.41 (1.18–1.69)	<0.001	1.23 (1.02–1.49)	0.032
Sex				
0	Reference		Reference	
1	1.27 (1.13–1.42)	<0.001	1.20 (1.07–1.35)	0.002
Smoking status				
Never	Reference		Reference	
Former	1.66 (1.47–1.87)	<0.001	1.53 (1.35–1.73)	<0.001
Current	1.88 (1.58–2.24)	<0.001	1.65 (1.38–1.98)	<0.001
Alcohol use status				
Never	Reference			
Former	0.97 (0.65–1.46)	0.884		
Current	0.77 (0.58–1.03)	0.079		
Overall health				
Excellent	Reference		Reference	
Good	1.46 (1.23–1.73)	<0.001	1.39 (1.16–1.65)	<0.001
General	1.96 (1.62–2.38)	<0.001	1.76 (1.43–2.17)	<0.001
Poor	2.77 (2.1–3.66)	<0.001	2.40 (1.78–3.25)	<0.001
BMI	1.01 (1.00–1.03)	0.031	0.99 (0.98–1.01)	0.409
Weekly light PA				
None or less than RT	Reference			
RT	0.94 (0.81–1.08)	0.379		
More than RT	1.11 (0.97–1.26)	0.126		
Weekly moderate PA				
None or less than RT	Reference		Reference	
RT	1.07 (0.92–1.23)	0.392	1.12 (0.96–1.30)	0.155
More than RT	1.20 (1.05–1.37)	0.009	1.17 (1.01–1.36)	0.038
Weekly vigorous PA				
None or less than RT	Reference		Reference	
RT	0.79 (0.67–0.93)	0.005	0.85 (0.72–1.01)	0.061
More than RT	0.96 (0.82–1.12)	0.583	0.98 (0.83–1.16)	0.840
Total weekly PA				
None or less than RT	Reference			
RT	0.86 (0.72–1.03)	0.108		
More than RT	0.87 (0.76–1.01)	0.061		
Yes	1.01 (0.9–1.13)	0.879		

CI, confidence interval; HR, hazard ratio; RT, PA duration recommended by the WHO.

In contrast, the risk factors for UC are more complex. Smoking, age, and health status are all closely associated with the occurrence of UC. The risk of the disease is significantly higher in men, individuals over 60 years old, and smokers. In addition, when the duration of moderate PA exceeds the recommended WHO guidelines, a trend of increased risk is observed (HR = 1.17, *p* = 0.038). These findings suggest that excessive participation in moderate PA may have a counterproductive effect on the prevention of UC.

### 3.2 Cumulative risk of PA and IBD incidence

**Figure 1:**
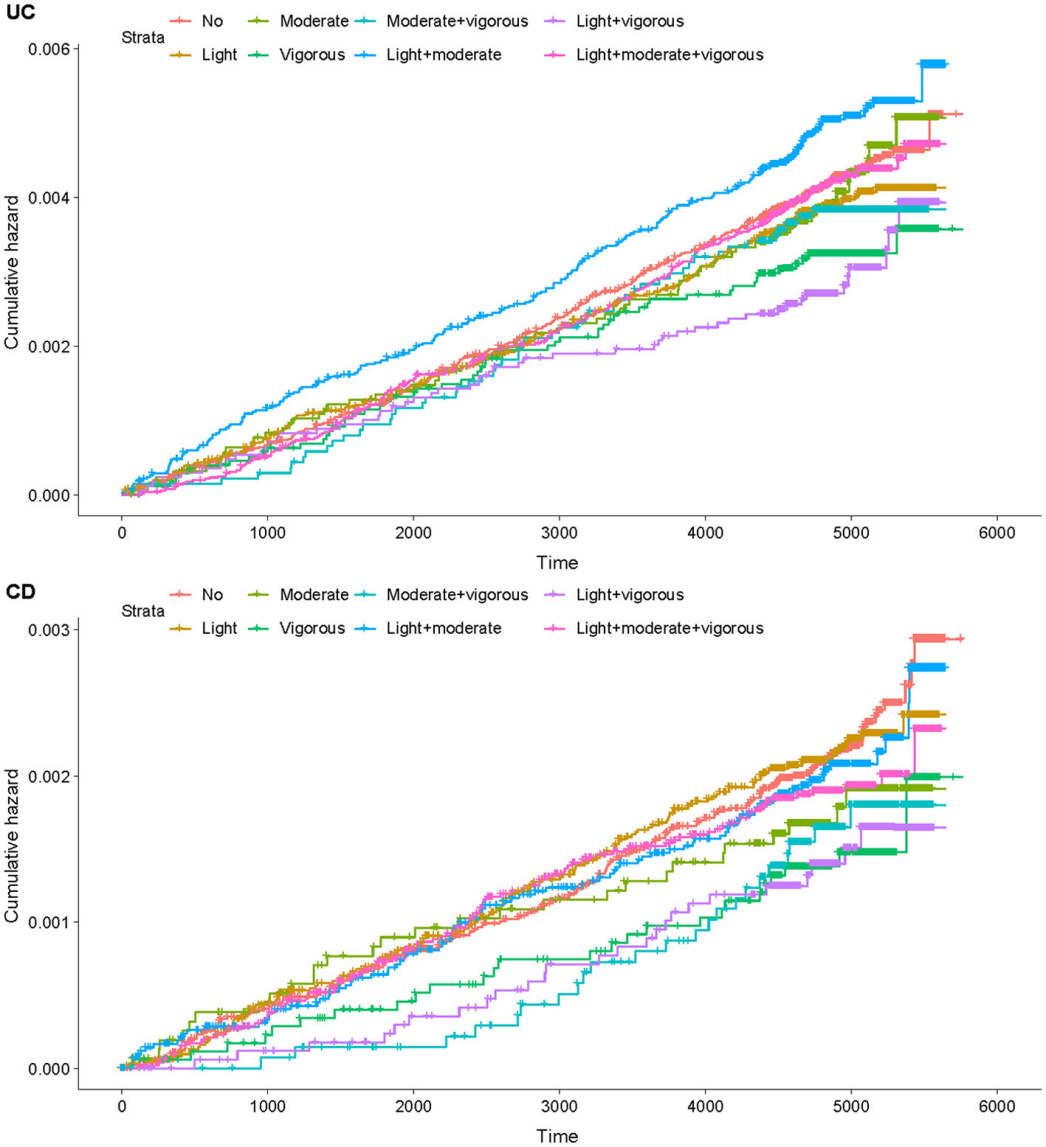
Cumulative risk curve of physical activity (PA) and inflammatory bowel disease (IBD) incidence.

Figure 1 presents the cumulative risk curves for incident CD and UC according to different PA patterns. According to Figure 1, participants who reported no PA or only engaged in light-intensity activity exhibited the highest cumulative hazards over the follow-up period. In contrast, those who engaged in moderate- or vigorous-intensity activities showed a lower cumulative hazard of CD. The most significant risk reduction was observed among participants who reported combinations of multiple intensities (e.g., moderate + vigorous, or light, moderate, and vigorous combined), with their curves consistently remaining at the lowest levels throughout the follow-up. Overall, the trajectories suggest a clear gradient: no or light activity was associated with the highest cumulative hazard of CD, followed by moderate or vigorous PA alone, while diverse activity profiles that combined multiple intensities were associated with the lowest cumulative risk.

However, the cumulative risk curve for UC incidence shows that, during the early follow-up period, the curves of each group overlapped significantly. Around the middle of the follow-up period, a clear difference emerged. These patterns do not support a monotonic dose–response relationship between activity intensity and UC risk. Instead, the associations appeared weak and heterogeneous, varying by PA pattern.

### 3.3 Nonlinear relationship between PA and IBD incidence

**Figure 2:**
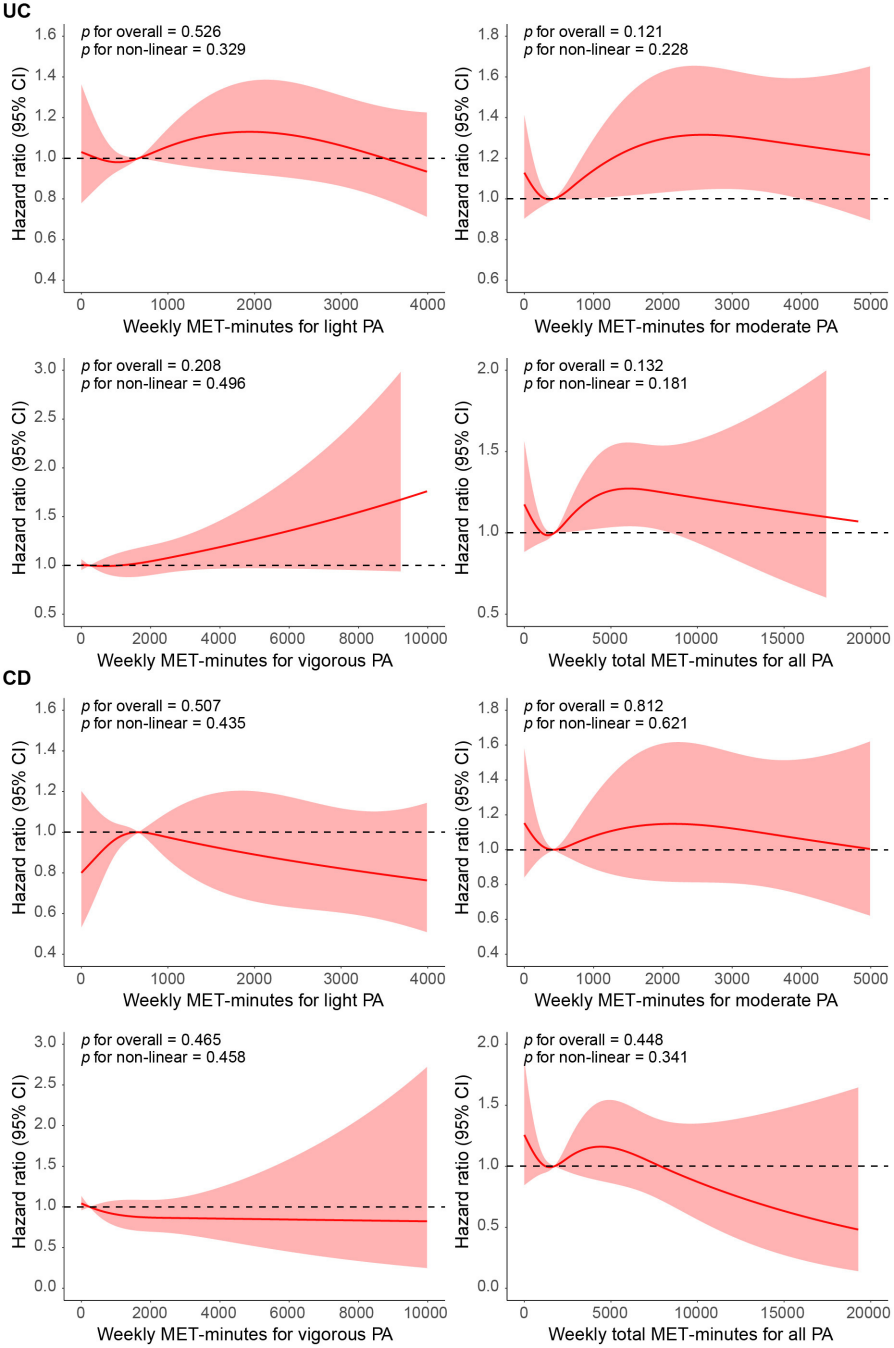
Restricted cubic spline (RCS) analysis of physical activity (PA) and inflammatory bowel disease (IBD) incidence.

According to Figure 2, although no statistically significant nonlinear relationship was found between CD, UC, and different intensities of PA (all *p*-values > 0.05), the RCS curves in the figure show trends suggesting potential protective effects. For CD, the curves for light and moderate PA demonstrate a slight downward trend, indicating that as the amount of PA increases, the risk of CD may decrease, although these changes did not reach statistical significance (*p* = 0.507 and *p* = 0.812, respectively). Similarly, for UC, the curves for light and moderate PA also show downward trends (*p* = 0.526 and *p* = 0.121, respectively), but these trends did not reach statistical significance either. Overall, while these results do not show strong statistical significance, the corresponding confidence intervals are relatively wide. The trend observed in the RCS curves suggests that participating in moderate PA may have a protective effect in reducing the risks of IBD.

### 3.4 Subgroup analyses

According to Tables 3, 4, subgroup analysis revealed that, in males, reaching the recommended level of light PA was associated with an increased prevalence of CD (HR = 1.43, *p* = 0.039). In contrast to the overall results, female participants who exceeded the total PA recommended by the WHO showed a significant protective effect against UC (HR = 0.60, *p* = 0.037). Notably, in the smoking subgroup, engaging in moderate PA for more than the recommended duration appeared to increase the risk of UC (HR = 1.35, *p* = 0.015), whereas exceeding the WHO-recommended total weekly PA time significantly reduced the risk of UC (HR = 0.57, *p* = 0.013). Overall, the subgroup analysis results of this study were generally consistent with the findings from the overall population data, but they also highlighted the potential moderating effects of gender and smoking status on the relationship between PA and disease risk.

**Table 3:** Subgroup analysis of physical activity (PA) and incidence of inflammatory bowel disease (IBD) stratified by sex.

Variables	All participants		Females		Males	
	HR (95% CI)	*p*	HR (95% CI)	*p*	HR (95% CI)	*p*
Crohn's disease						
Total weekly PA						
None or less than RT	Reference		Reference		Reference	
RT	1.02 (0.79–1.32)	0.855	0.71 (0.42–1.19)	0.190	1.14 (0.64–2.01)	0.663
More than RT	0.98 (0.74–1.29)	0.873	0.74 (0.40–1.36)	0.332	0.88 (0.45–1.72)	0.705
Weekly light PA						
None or less than RT	Reference		Reference		Reference	
RT	NA	NA	0.85 (0.60–1.21)	0.374	1.43 (1.02–2.02)	0.039
More than RT	NA	NA	0.91 (0.62–1.34)	0.640	1.31 (0.90–1.90)	0.162
Weekly moderate PA						
None or less than RT	Reference		Reference		Reference	
RT	NA	NA	1.38 (0.75–2.53)	0.299	1.15 (0.73–1.83)	0.547
More than RT	NA	NA	1.58 (0.87–2.88)	0.137	0.90 (0.56–1.45)	0.654
Weekly vigorous PA						
None or less than RT	Reference		Reference		Reference	
RT	0.97 (0.73–1.29)	0.844	1.12 (0.71–1.76)	0.636	0.99 (0.64–1.53)	0.962
More than RT	1.09 (0.82–1.44)	0.546	0.97 (0.62–1.52)	0.903	1.23 (0.84–1.81)	0.293
**Ulcerative colitis**						
Total weekly PA						
None or less than RT	Reference		Reference		Reference	
RT	NA	NA	0.76 (0.50–1.14)	0.182	0.79 (0.54–1.16)	0.229
More than RT	NA	NA	0.60 (0.37–0.97)	0.037	0.66 (0.42–1.03)	0.068
Weekly light PA						
None or less than RT	Reference		Reference		Reference	
RT	NA	NA	1.06 (0.71–1.57)	0.785	1.05 (0.83–1.34)	0.676
More than RT	NA	NA	1.11 (0.75–1.65)	0.591	1.33 (1.04–1.70)	0.025
Weekly moderate PA						
None or less than RT	Reference		Reference		Reference	
RT	1.12 (0.96–1.30)	0.155	1.38 (0.75–2.53)	0.299	1.12 (0.81–1.57)	0.487
More than RT	1.17 (1.01–1.36)	0.038	1.58 (0.87–2.88)	0.137	1.13 (0.81–1.57)	0.460
Weekly vigorous PA						
None or less than RT	Reference		Reference		Reference	
RT	0.85 (0.72–1.01)	0.061	0.79 (0.57–1.08)	0.141	0.93 (0.69–1.24)	0.604
More than RT	0.98 (0.83–1.16)	0.840	0.95 (0.71–1.26)	0.714	1.03 (0.80–1.33)	0.831

CI, confidence interval; HR, hazard ratio; RT, PA duration recommended by the WHO.

**Table 4:** Subgroup analysis of physical activity (PA) and incidence of inflammatory bowel disease (IBD) stratified by smoking status.

Variables	All participants		Never smokers		Smokers	
	HR (95% CI)	*p*	HR (95% CI)	*p*	HR (95% CI)	*p*
Crohn's disease						
Total weekly PA						
None or less than RT	Reference		Reference		Reference	
RT	1.02 (0.79–1.32)	0.855	1.04 (0.58–1.85)	0.905	0.76 (0.46–1.27)	0.297
More than RT	0.98 (0.74–1.29)	0.873	1.04 (0.53–2.04)	0.920	0.61 (0.34–1.13)	0.115
Weekly light PA						
None or less than RT	Reference		Reference		Reference	
RT	NA	NA	1.37 (0.97–1.94)	0.077	0.89 (0.63–1.26)	0.518
More than RT	NA	NA	0.97 (0.66–1.44)	0.882	1.22 (0.85–1.76)	0.288
Weekly moderate PA						
None or less than RT	Reference		Reference		Reference	
RT	NA	NA	0.99 (0.63–1.55)	0.969	1.05 (0.63–1.76)	0.852
More than RT	NA	NA	1.10 (0.73–1.66)	0.637	1.04 (0.62–1.73)	0.886
Weekly vigorous PA						
None or less than RT	Reference		Reference		Reference	
RT	0.97 (0.73–1.29)	0.844	1.12 (0.71–1.76)	0.636	1.09 (0.70–1.70)	0.699
More than RT	1.09 (0.82–1.44)	0.546	0.97 (0.62–1.52)	0.903	1.16 (0.77–1.74)	0.474
**Ulcerative colitis**						
Total weekly PA						
None or less than RT	Reference		Reference		Reference	
RT	NA	NA	0.89 (0.58–1.34)	0.570	0.69 (0.47–1.01)	0.057
More than RT	NA	NA	0.70 (0.43–1.14)	0.155	0.57 (0.37–0.89)	0.013
Weekly light PA						
None or less than RT	Reference		Reference		Reference	
RT	NA	NA	1.02 (0.79–1.32)	0.876	1.01 (0.80–1.28)	0.937
More than RT	NA	NA	1.02 (0.77–1.34)	0.908	1.35 (1.06–1.73)	0.015
Weekly moderate PA						
None or less than RT	Reference		Reference		Reference	
RT	1.12 (0.96–1.30)	0.155	1.08 (0.75–1.56)	0.687	1.12 (0.79–1.59)	0.525
More than RT	1.17 (1.01–1.36)	0.038	1.14 (0.79–1.65)	0.490	1.12 (0.79–1.59)	0.511
Weekly vigorous PA						
None or less than RT	Reference		Reference		Reference	
RT	0.85 (0.72–1.01)	0.061	0.95 (0.69–1.32)	0.764	0.80 (0.60–1.06)	0.113
More than RT	0.98 (0.83–1.16)	0.840	1.10 (0.82–1.48)	0.510	0.91 (0.71–1.17)	0.460

CI, confidence interval; HR, hazard ratio; RT, PA duration recommended by the WHO.

### 3.5 Sensitivity analysis

Overall, according to Table 5, the results of the sensitivity analysis support the conclusions drawn from the total population data, indicating that the results remain robust after excluding the relevant groups. This is particularly true for the analysis of CD. However, the relationship between the incidence of UC and activity intensity is more complex. After excluding participants with a disease duration of less than 3 years, the association between exceeding the recommended total PA time and the risk of UC became relatively significant (HR = 0.59, *p* = 0.004). For individuals with a BMI within the healthy range, exceeding the recommended total PA demonstrated a significant protective effect (HR = 0.60, *p* = 0.01). However, after engaging in excessive light PA, there was a trend toward an increased risk (HR = 1.27, *p* = 0.022). This trend is consistent with the findings from our subgroup analysis for the male and smoker groups.

**Table 5:** Sensitivity analysis of physical activity (PA) and incidence of inflammatory bowel disease (IBD).

Variables	All participants		18.5<BMI<30 group		Excluding early incidence (<3 years)	
	HR (95% CI)	*p*	HR (95% CI)	*p*	HR (95% CI)	*P*
Crohn's disease						
Total weekly PA						
None or less than RT	Reference		Reference		Reference	
RT	1.02 (0.79–1.32)	0.855	0.73 (0.46–1.15)	0.170	0.75 (0.49–1.15)	0.188
More than RT	0.98 (0.74–1.29)	0.873	0.62 (0.36–1.05)	0.078	0.74 (0.45–1.22)	0.237
Weekly light PA						
None or less than RT	Reference		Reference		Reference	
RT	NA	NA	1.06 (0.80–1.41)	0.700	1.16 (0.88–1.52)	0.290
More than RT	NA	NA	1.09 (0.80–1.48)	0.591	1.09 (0.81–1.47)	0.582
Weekly moderate PA						
None or less than RT	Reference		Reference		Reference	
RT	NA	NA	1.07 (0.71–1.61)	0.737	1.08 (0.72–1.62)	0.696
More than RT	NA	NA	1.03 (0.68–1.55)	0.895	1.12 (0.75–1.67)	0.594
Weekly vigorous PA						
None or less than RT	Reference		Reference		Reference	
RT	0.97 (0.73–1.29)	0.844	1.00 (0.69–1.43)	0.984	1.01 (0.71–1.44)	0.945
More than RT	1.09 (0.82–1.44)	0.546	1.18 (0.85–1.64)	0.320	1.17 (0.85–1.61)	0.331
**Ulcerative colitis**						
Total weekly PA						
None or less than RT	Reference		Reference		Reference	
RT	NA	NA	0.81 (0.58–1.12)	0.199	0.70 (0.51–0.95)	0.023
More than RT	NA	NA	0.60 (0.41–0.88)	0.010	0.59 (0.41–0.85)	0.004
Weekly light PA						
None or less than RT	Reference		Reference		Reference	
RT	NA	NA	1.01 (0.82–1.23)	0.956	0.93 (0.76–1.12)	0.434
More than RT	NA	NA	1.27 (1.04–1.56)	0.022	1.10 (0.90–1.34)	0.369
Weekly moderate PA						
None or less than RT	Reference		Reference		Reference	
RT	1.12 (0.96–1.30)	0.155	1.08 (0.82–1.44)	0.579	1.26 (0.95–1.67)	0.113
More than RT	1.17 (1.01–1.36)	0.038	1.15 (0.87–1.52)	0.331	1.27 (0.96–1.68)	0.101
Weekly vigorous PA						
None or less than RT	Reference		Reference		Reference	
RT	0.85 (0.72–1.01)	0.061	0.89 (0.70–1.13)	0.341	0.96 (0.75–1.21)	0.708
More than RT	0.98 (0.83–1.16)	0.840	0.95 (0.76–1.17)	0.607	1.15 (0.93–1.42)	0.196

BMI, body mass index; CI, confidence interval; HR, hazard ratio; RT, PA duration recommended by the WHO.

## 4 Discussion

This prospective cohort study aimed to elucidate the association between PA and the long-term incidence of IBD, specifically investigating the dose-response effects of varying PA intensities on the onset of CD and UC. By leveraging a substantial dataset of 293,578 individuals and employing rigorous statistical frameworks—including multivariable Cox proportional hazards regression, cumulative incidence curves, and RCS analysis—several clinically significant findings have emerged.

Our analysis demonstrates that higher levels of PA are consistently associated with a reduced risk of developing CD. In contrast, the relationship between PA and UC appears more nuanced; while a general protective effect was not observed across the entire cohort, a significant association was identified within specific subpopulations and at higher intensity thresholds. These findings suggest that the impact of PA on IBD pathogenesis is characterized by significant heterogeneity, with the protective benefits highly dependent on activity intensity and individual demographic profiles.

Furthermore, the sensitivity and subgroup analyses performed in this study reinforce the stability of these associations, providing a more granular understanding of how lifestyle modifications might influence IBD risk across different patient phenotypes.

It is worth noting that because the data on PA and related covariates were only collected at the baseline period, we were unable to consider the longitudinal changes in activity levels during the follow-up period. Given the evidence suggesting that the protective benefits of exercise are cumulative and may depend on the lifelong PA pattern, the baseline indicators used here may not fully reflect the lifelong PA exposure of the participants, and cannot assess the impact of changes in PA.

Another notable limitation is that the risk of developing IBD is influenced by multiple interacting factors. Dietary patterns and drug exposure (such as nonsteroidal anti-inflammatory drugs or antibiotics) are important modifiable risk factors. However, our analytical model did not include potential confounding factors. Due to the lack of detailed longitudinal data on these variables, they could not be incorporated as covariates in the multivariate model. Therefore, the possibility of residual confounding factors cannot be excluded; that is, unmeasured dietary habits or drug use may have affected the observed association between PA and the incidence of IBD.

Our analysis revealed a significant inverse association between PA and the risk of CD. Specifically, individuals who exceeded the total weekly PA levels recommended by the WHO exhibited a 22% lower risk of developing CD compared to those who failed to meet these benchmarks. Intensity-specific analysis further demonstrated that meeting the WHO-recommended range for vigorous PA alone was associated with a 23% risk reduction.

Interestingly, the lowest cumulative risk for CD was observed in participants who engaged in a combination of PA intensities (e.g., light + vigorous or light + moderate) within the recommended weekly range. This suggests a potential synergistic effect of multimodal exercise. Diversifying activity intensities may optimize physiological resilience through complementary pathways: moderate-intensity aerobic exercise is known to enhance basal metabolic regulation, while high-intensity PA significantly improves cardiopulmonary endurance and systemic immune surveillance.

These findings for CD are largely consistent with established literature. A meta-analysis by Wang et al. (10) reported that high levels of PA were associated with a 37% reduction in CD risk compared to sedentary or low-activity cohorts. Similarly, data from the Nurses' Health Study I and II indicated that women engaging in >27 MET-hours per week—roughly equivalent to 9 hours of brisk walking—experienced a 44% lower risk of CD compared to those with minimal activity (6).

Our results also echo earlier case-control findings, such as those by Persson et al. (11), which identified a negative correlation between regular PA and CD risk, while noting no such association for UC. By utilizing the large-scale UK Biobank cohort, our study reinforces these historical observations with contemporary, high-powered longitudinal data, confirming that consistent PA is a robust protective factor against CD.

The inverse association between PA and the risk of CD is supported by several compelling biological mechanisms.

First, regular PA has been shown to upregulate autophagy, a critical cellular degradation and recycling process (12). Clinical evidence suggests that impaired autophagy is a significant risk factor for the pathogenesis of IBD, with specific genetic polymorphisms related to autophagic pathways being more strongly linked to CD than to UC (13). By stimulating these pathways, exercise may enhance intestinal epithelial integrity and intracellular pathogen clearance.

Additionally, PA exerts a potent systemic and local anti-inflammatory effect. *In vivo* studies using murine models have demonstrated that PA can attenuate intestinal inflammatory responses and confer mucosal protection by downregulating pro-inflammatory cytokines and apoptotic proteins (14, 15). By modulating the cytokine profile and reducing oxidative stress, PA may raise the threshold for the clinical manifestation of symptomatic IBD.

Furthermore, the role of PA in mental health regulation cannot be overlooked (16, 17). Psychological distress is increasingly recognized as a modifiable risk factor for CD onset. Data from the Nurses' Health Study cohorts revealed that women with significant depressive symptoms—defined by a Mental Health Inventory (MHI-5) score of <52—exhibited a markedly increased risk of developing CD (18).

Given that regular exercise is an established intervention for reducing anxiety and depression, its protective effect against CD may be partially mediated through the gut-brain axis, mitigating the pro-inflammatory physiological states associated with chronic stress. Collectively, these multifaceted pathways provide a robust biological rationale for the protective role of PA in CD development.

In contrast to the findings for CD, the relationship between PA and UC appears significantly more complex. Our research identified that engaging in moderate PA for durations exceeding the WHO-recommended thresholds was associated with a 17% increased risk of developing UC. Interestingly, a distinct trend emerged within specific subgroups—namely males, smokers, and individuals within a normal BMI range. In these populations, exceeding the total recommended PA time was associated with a reduced risk of UC; however, exceeding the recommended duration for light PA specifically was associated with an increased risk. While moderate and vigorous PA showed a marginal trend toward increased risk, these did not reach statistical significance.

Although several landmark studies have failed to identify a significant association between PA and UC risk (6, 10, 11), our findings align with a large Japanese cohort study which reported that the risk of UC increased in correlation with occupational PA. In that study, manufacturing and service-oriented roles—characterized by prolonged, repetitive, and often low-intensity movements—were classified as medium-to-high occupational PA. This phenomenon, often termed the “PA Paradox,” suggests that high levels of workplace PA may paradoxically lead to adverse health outcomes, particularly in men, whereas vigorous leisure-time PA remains protective (19). The mechanisms underlying this paradox may involve the non-sustainable nature of prolonged, low-intensity activity without adequate recovery. Such exertion can lead to sustained elevations in 24-h heart rate and blood pressure, potentially inducing a state of chronic physiological stress (20, 21).

The adverse effects of excessive PA duration on colonic health are further supported by experimental evidence. Prolonged and strenuous PA has been shown to exacerbate colonic mucosal damage in murine models of induced colitis (22). Potential pathways include microcirculatory compromise and oxidative stress. High-volume or repetitive PA may reduce mesenteric and mucosal microcirculation, whereas excessive durations of activity may trigger an imbalance in oxidative stress markers within the colonic mucosa.

These mechanisms may explain why exceeding light PA thresholds was associated with an increased risk of UC among men and smokers in our cohort. We cannot exclude the possibility that these paradoxical effects are driven by the cumulative physiological strain of prolonged, repetitive light-to-moderate activity, which lacks the anti-inflammatory benefits typically associated with structured, high-intensity exercise.

Our analysis also indicated that achieving the total weekly PA duration recommended by the WHO may exert a protective effect against UC within specific demographics, specifically in females, smokers, and individuals with a normal BMI. This suggests that while the relationship between PA and UC is generally complex, certain populations may still derive significant preventative benefits from adherence to established activity guidelines. These findings are consistent with two recent studies showing that longer durations of the accelerometer-measured moderate-to-vigorous PA (MVPA) were associated with a lower risk of intestinal resection and reduced mortality rate (23). Additionally, another study found that structured PA interventions for IBD patients did not report disease relapses during most of the intervention period, suggesting that structured PA measures did not exacerbate the disease. However, due to the differences in the type and duration of the PA interventions, the optimal type of PA remains undetermined. Nevertheless, existing studies have generally reported beneficial effects of PA (24). This study mainly included IBD patients who were in remission or had a mild disease activity (75%), complementing prior research. Because patients with mild symptoms of IBD (especially those with UC) are usually managed in outpatient settings and may lack comprehensive hospital records. Therefore, relying solely on hospitalization data may ultimately lead to bias the findings toward associations between PA and severe or hospitalized UC.

This type of selection bias can directly affect the estimation of physiological indicators, such as heart rate. In particular, heart rate is a key clinical indicator for assessing the severity of UC. Patients with severe UC often have a pronounced systemic inflammatory response, manifested by high fever, anemia, and tachycardia (rapid pulse). Therefore, the average heart rate in hospitalized patients with severe disease is likely to increase. In contrast, mild UC patients usually do not have systemic symptoms, and their heart rate generally remains within the normal range. Notably, this imbalance may lead to an overestimation of the resting heart rate and an underestimation of heart rate recovery (HRR). As a result, findings may be misinterpreted as evidence of widespread cardiac autonomic nerve dysfunction in IBD patients (especially UC patients), thereby masking the preserved recovery ability associated with regular PA.

Although the optimal type of PA for improving health outcomes in UC patients remains unclear, existing studies have shown that moderate and reasonable physical activity is associated with certain health benefits.

This positive association between PA and UC may be partly related to the anti-inflammatory effects of PA. Several biological mechanisms support the protective role of PA in colonic health. One long-standing hypothesis suggests that regular exercise correlates with increased hydration and water intake, which has been associated with a reduction in the prevalence of colorectal lesions (25). Beyond hydration, growing evidence highlights the role of PA in modulating the intestinal microbiota. Regular exercise promotes a more diverse and resilient microbial composition, which enhances mucosal immunity and metabolic homeostasis (26, 27). A recent study identified 25 key metabolites related to MVPA. These metabolite characteristics reflect multiple beneficial changes, such as optimized lipoprotein profiles, reduced fatty acid saturation, enhanced energy metabolism, and decreased systemic inflammation levels. Additionally, there was an interaction between PA and the genetic risk of IBD (28). Specifically, PA has been shown to promote muscle-organ crosstalk. Exercise-induced muscle contractions release myokines that exert systemic anti-inflammatory effects. Furthermore, PA decreases visceral fat tissue, thereby reducing the systemic secretion of pro-inflammatory adipokines. Moreover, physical exertion increases the abundance of beneficial, butyrate-producing bacteria, which are essential for maintaining the colonic epithelial barrier (29).

These integrated pathways—ranging from fat tissue reduction to the stabilization of the intestinal microbiome—collectively contribute to the protective role of PA against the development of IBD (30).

Based on existing research results and public health recommendations, the optimal amount of PA for preventing IBD may require a balance between the total PA duration and adequate recovery time, while emphasizing the diversity and a combination of different PA intensities. Because IBD itself and hormone therapy can increase the risk of osteoporosis, engaging in moderate weight-bearing PA and resistance training can effectively increase bone density. For patients with IBD in the remission or with mild active phases, it is recommended to engage in the light to moderate PA at levels recommended by the WHO for the specified duration. For example, this may include 15–45 min of walking per day; daily moderate stretching exercises; 1–3 sessions per week of gentle yoga or Tai Chi to support the nervous system and alleviate anxiety and depressive symptoms; along with 1–3 sessions per week of aerobic PA, such as swimming or brisk walking, which can enhance the cardiopulmonary function, while avoiding excessive abdominal strain. For patients with moderate-to-severe active IBD, MVPA should be avoided. Instead, gentle stretching exercises in bed, along with deep breathing and relaxation training, may be more appropriate.

### 4.1 Strengths and limitations

This study has several methodological strengths. First, the prospective cohort design minimizes selection, recall, and protopathic biases. Second, the UK Biobank registry ensured high participant retention with minimal loss to follow-up. Third, as PA metrics and covariates were documented at baseline prior to IBD diagnosis, we established a clear temporal sequence, ensuring activity levels were not influenced by pre-existing symptoms. Fourth, PA assessment followed standardized protocols: self-reported data were converted to MET scores according to IPAQ guidelines, and intensity stratification aligned with WHO thresholds, ensuring reliable and valid exposure variables. In addition, our study included different levels of PA and conducted separate analyses for UC and CD. In contrast, many previous studies have not differentiated between UC and CD or have focused on a single level of PA intensity (e.g., occupational PA or MVPA). Therefore, our research provides a more nuanced understanding of the impacts of various PA intensities in patients with UC and CD.

Despite its strengths, this study has several limitations that warrant consideration. First, data regarding PA and relevant covariates were collected exclusively at baseline. Consequently, we cannot account for potential longitudinal changes in activity levels during the follow-up period. This may introduce exposure misclassification bias. Second, the demographic profile of the UK Biobank cohort presents a potential age-related bias. With a baseline mean age of 55 years—compared with the typical IBD peak incidence of 30–40 years—our findings may reflect a “late-onset” population with distinct risk factors. This demographic characteristic may also affect the reliability of self-reported PA. Finally, the “healthy volunteer” effect inherent to the UK Biobank may limit the generalizability of our results to more sedentary or socioeconomically diverse populations.

Furthermore, the assessment of PA in this study was based on total MET scores, which did not differentiate between the specific types of PA activities within each intensity group (e.g., swimming, cycling, or weightlifting). This might obscure the different physiological or immunological effects associated with different types of PA. Future research incorporating objective measures, such as accelerometry combined with detailed activity logs, is necessary to further refine these associations and enhance the practical application of PA recommendations in IBD prevention.

Finally, our analytical model did not incorporate all potential confounding factors (e.g., dietary patterns, NSAID use, and antibiotic exposure). This could lead to residual confounding. Future prospective research should aim to integrate comprehensive nutritional assessments and medication registries to further isolate the independent effect of PA and clarify its role within the broader landscape of IBD environmental risk factors.

## 5 Conclusion

In this large-scale prospective cohort study leveraging the UK Biobank, PA—particularly multimodal regimens combining different intensities—was significantly associated with a reduced risk of CD. While these findings establish a robust inverse association with disease incidence, it remains to be determined whether therapeutic exercise can similarly modify the clinical course of patients with established CD; further interventional research is warranted to clarify this distinction. The relationship between PA and UC is notably more complex and heterogeneous. While moderate PA appears to offer a protective benefit within specific subgroups (males, smokers, and individuals with a normal BMI), excessive light-intensity PA or prolonged, monotonous moderate-to-low intensity exertion may paradoxically increase UC risk. This phenomenon supports the existence of a “PA paradox,” where the benefits of exercise are contingent upon the specific balance of intensity and duration.

## Data Availability

The original contributions presented in the study are included in the article/Supplementary material, further inquiries can be directed to the corresponding author.
